# Systematic Analysis of SH-SY5Y Differentiation Protocols and Neuronal Subtype Abundance

**DOI:** 10.1007/s10571-025-01627-0

**Published:** 2025-11-15

**Authors:** Marina Prisacar, Svenja Esser, Maximilian Hausherr, Bilhan Karacora, Yulia Vyushkova, Martin Eisenacher, Robin Grugel, Katrin Marcus, Britta Eggers

**Affiliations:** 1 Medizinisches Proteom-Center, Medical Faculty, Ruhr-University Bochum, 44801 Bochum, Germany; 2 Medical Proteome Analysis, Center for Protein Diagnostics (PRODI), Ruhr-University Bochum, 44801 Bochum, Germany; 3 Core Unit for Bioinformatics (CUBiMed.RUB), Medical Faculty, Ruhr-University Bochum, 44801 Bochum, Germany

**Keywords:** SH-SY5Y, Differentiation, Neuronal subtype, Proteomics, Mass spectrometry

## Abstract

The SH-SY5Y cell line is a triple-cloned subline of SK-N-SH cells originally isolated in the early 1970s from a bone marrow biopsy of a four-year-old female patient suffering from neuroblastoma. Since then, this cell line has been used as one of the major cell culture models in neuroscience and to study neurodegeneration, as it comprises many of the biochemical and functional properties of neural precursor cells. Differentiation of neuronal precursor cells into a more mature phenotype represents one of the key steps and directed differentiation utilising various reagents is thought to provoke a defined neuronal subtype. Unfortunately, until now there is no consensus, which protocol shall be utilised to reach a specific neuronal subtype. Thus, the aim of the present work was to evaluate four common standard protocols for the differentiation of SH-SY5Y cells and to investigate the respective influences of varying parameters of these differentiation strategies. For this purpose, morphological analyses, mass spectrometry-based quantification of specific marker proteins, time-course protein expression profiling and global proteomics were conducted. On the level of morphology a low serum concentration favoured the abundance of mature neuronal cells containing long and branched neurites. Further low serum levels favoured the expression of dopaminergic marker proteins, in particular DDC, especially when utilising retinoic acid as differentiation agent. Our study clearly shows that an a priori characterisation of SH-SY5Y cells is indispensable to assess the abundance of neuronal subtypes and by that to ensure that the utilised differentiation approach is appropriately aligned with the specific research question.

**Supplementary Information:**

The online version contains supplementary material available at 10.1007/s10571-025-01627-0.

## Background

The most commonly used cell line to study neuronal function and dysfunction is the human neuroblastoma cell line SH-SY5Y, one of the few immortalised neuronal cell lines of fully human origin (Ioghen et al. 2023) and is therefore frequently used in the field of neuroscience, in particular to study neurodegeneration and to mimic neurodegenerative diseases in vitro. The SH-SY5Y cell line is a triple-cloned subline of SK-N-SH cells that were originally isolated in the early 1970s from a bone marrow biopsy of a four-year-old female patient suffering from neuroblastoma (Biedler et al. 1973). Since then, this cell line has been used as a model for neurons, as it has many of the biochemical and functional properties of neuronal cells.

Undifferentiated (undiff) SH-SY5Y cells show a continuously high proliferation rate, tend to grow in clusters and develop few short processes. This indicates a phenotype that resembles a neoplastic state or a cancerous cell type (Ioghen et al. 2023; Kovalevich and Langford 2013). To convert SH-SY5Y cells into an advanced in vitro model directed differentiation into a specific neuronal subtype can be induced by adding various reagents to the cell culture medium (Xie et al. 2010; Cetin et al. 2022). These differentiation reagents include, for example, trans-retinoic acid (RA), phorbol esters such as 12-O-tetradecanoylphorbol-13-acetate (TPA), neurotrophins, such as brain-derived neurotrophic factor, dibutyryl-cAMP, purine or staurosporine. Among these, RA or the combination of RA and TPA is the most commonly applied strategy in research as already stated in a comprehensive review (Xicoy et al. 2017).

Differentiation further involves a number of specific events, including the formation and expansion of neuritic projections, increased electrical excitability of the plasma membrane, the formation of functional synapses and the expression of mature neuronal markers, showing similarity to living neurons of the human CNS (Ioghen et al. 2023; Kovalevich and Langford 2013; Xie et al. 2010; Cetin et al. 2022).

Unfortunately, only few studies verified a successful differentiation of cells, or further verified a specific neuronal subtype within their culture (Xicoy et al. 2017). However, depending on the research question, this validation often represents an essential prerequisite, e.g., when investigating neurodegenerative diseases, such as Parkinson’s disease (PD) in which dopaminergic neurons are particularly vulnerable. Until today, it remains controversial which specific neuronal subtype is ultimately mediated by the frequently used differentiation methods (Kovalevich and Langford 2013; Xicoy et al. 2017; Korecka et al. 2013). However, a systematic review of scientific publications on SH-SY5Y cells utilised in PD research showed that of 962 papers included only 16% did differentiate, with various differentiation agents being used to achieve a dopaminergic phenotype (Ioghen et al. 2023; Xicoy et al. 2017; Lopes et al. 2017). However, solely 6% of publications verified the presence of dopaminergic markers utilizing different approaches stressing the need to comprehensively characterise the outcome of the various differentiation approaches applied.

Thus, the aim of the present work was to validate the four most commonly followed standard protocols (RA only, RA/TPA, high/low FBS) for the differentiation of SH-SY5Y cells to investigate the respective influences of varying parameters in respect to the induction of a specific neuronal subtype. In addition, morphological changes indicative of mature neuronal cells, resulting over the course of differentiation, were investigated, to verify an overall successful differentiation. For that, immunocytochemistry and mass spectrometry-based identification and quantification of specific marker proteins, tracking of time-dependent protein abundance profiles and global proteomic characterisation was performed. With this we aimed to provide a resource for researches aiming to utilize SH-SY5Y cells in their work, to ensure that prerequisites, such as the presence of mature neuronal cells, specific neuronal subtypes or essential marker proteins, are met and aligned with their specific research question.

## Methods

### Cell Culture

The SH-SY5Y cell line was obtained from the Leibnitz Institute DSMZ-German Collection of Microorganisms and Cell Cultures GmbH (Braunschweig, Germany). For all experiments, early passages (P7 to P11) were used, since it is thought, that SH-SY5Y cells may start to lose their neuronal characteristics and the potential to generate neurites in passages equal or greater than P20. The undifferentiated, wild-type cells were maintained in standard growth medium for cultivation (DMEM, 10% FBS, 1% Glutamine, 1% Penicillin/Streptomycin, all PAN Biotech, Aidenbach, Germany) and grown in the presence of 5% CO2 in an incubator (HeraCell 150i, ThermoFisher Scientific, Darmstadt, Germany) at 37 °C. The growth medium was changed every third day.

Cells were differentiated at a confluence of approx. 80%. The differentiation media were always prepared on the same day, as RA in particular is unstable when exposed to light and oxygen. The media composition of all four differentiation strategies is outlined in Table 1. For each differentiation strategy three biological replicates per time point (day 1-day 6) were prepared, as well as six biological replicates of undifferentiated cells.

**Table 1:** Media composition of the tested differentiation strategies

Differentiation strategy	Media composition day1-day3	Media composition day 4-day 6
High RA/TPA	DMEM, 10% FBS, 1% Glutamine, 1% Penicillin/Streptomycin, 10 µM RA	DMEM, 10% FBS, 1% Glutamine, 1% Penicillin/Streptomycin, 80 nM TPA
Low RA/TPA	DMEM, 3% FBS, 1% Glutamine, 1% Penicillin/Streptomycin, 10 µM RA	DMEM, 3% FBS, 1% Glutamine, 1% Penicillin/Streptomycin, 80 nM TPA
High RA	DMEM, 10% FBS, 1% Glutamine, 1% Penicillin/Streptomycin, 10 µM RA	DMEM, 10% FBS, 1% Glutamine, 1% Penicillin/Streptomycin, 10 µM RA
Low RA	DMEM, 3% FBS, 1% Glutamine, 1% Penicillin/Streptomycin, 10 µM RA	DMEM, 3% FBS, 1% Glutamine, 1% Penicillin/Streptomycin, 10 µM RA

### Immunocytochemistry

30,000 cells were grown and differentiated in 24 wells containing glass cover slips covered with 0.1% gelatine (Sigma Aldrich, Taufkirchen, Germany). Fully differentiated cells were washed with sterile PBS and fixated in 4% paraformaldehyde for 30 min at RT. Cover slips were subsequently washed with sterile PBS and stored at 4 °C until further processing. For immunocytochemistry, cells were blocked with 4% BSA in sterile PBS for one hour. at RT. Primary antibodies were incubated over night at 4 °C see table 2 and secondary antibodies were incubated at RT the following day see table 3. Cover slips were mounted on ultrafrost glas slides with mounting media containing DAPI (VECTASHIELD Antifade Mounting Medium with DAPI, Biozol, Hamburg Germany).

**Table 2:** Information on primary antibodies, including dilution, vendor and Lot number

Primary Antibodies	Species	Dilution	Vendor (product number)	Lot-number
Vimentin (VIM)	Mouse	1:100	Santa Cruz Biotechnology, Texas, USA (sc-6260)	J1222
βIII-Tubulin (TUBB)	Rabbit	1:200	Abcam, Cambridge, UK (ab 6046)	GR171947-1
Dopamine	Rabbit	1:250	Immunosmol (IS1005)	140301

**Table 3:** Information on secondary antibodies, including dilution, vendor and Lot number

Secondary antibodies / Dyes	Species	Dilution	Vendor	Lot-number
Alexa Fluor™ 555	Anti-rabbit	1:1000	Invitrogen, Darmstadt, Deutschland (A21428)	1511349
Alexa Fluor™ 647	Anti-rabbit	1:1000	Invitrogen, Darmstadt, Deutschland (A21245)	2442141
Cy™2	Anti-mouse	1: 200	Jackson Immuno Research (115–225-205)	130192
DAPI	-	-	BIOZOL Diagnostica Vertrieb GmbH, Eching, Deutschland (VEC-H-1200)	ZG0602

### Determination of the Cell Subtype Distribution within the Different Cell Populations

The Olympus VS120 BX61VS virtual slide microscope and OlyVIA software (version 2.9) were used for fluorescence imaging. The fixed cells were assessed at 20 × object magnification. Five 500 × 500 μm sections per condition, distributed throughout the cell population, were captured for manual counting of subtypes that showed immunoreactivity to the βIII-tubulin (TUBB) and vimentin (VIM) antibodies. Cell counting was performed in the image processing software Fiji (ImageJ) (Schindelin et al. 2012) to determine the relative proportion of TUBB- and VIM-positive cells from the total number of cell bodies labelled with DAPI (approx. 430 to 460 cells per differentiation method). Descriptive statistics (mean values of relative abundances, standard deviation and standard error) and data visualisation were carried out in the program OriginPro 2023 (V10.0.0.154).

### Neurite Tracing

To assess neurite outgrowth, the projections of 100 TUBB-positive cells per condition were measured utilising ImageJ and the Fiji software extension, NeuronJ (Meijering et al. 2004; Meijering 2010). In preparation for neurite tracking in NeuronJ, the exported images were first processed, by converting them into an 8-bit version and duplicated. One of the images was inverted and the cell bodies with fully imaged neurites were marked, counted and measured. In the second duplicate, the brightness and contrast were adjusted and the background subtracted in order to remove any interfering artefacts before neurite tracking. This image was further processed in NeuronJ to measure primary, secondary and tertiary projections. The obtained data (total length of neurites, maximum neurite length and number of neurites) was exported and the total length and the number of neurites of were normalised to the number of analysed cells (*n* = 100). The statistical evaluation was carried out in OriginPro and a one-way ANOVA was applied. Significant effects within the differently treated cells were determined using the Bonferroni-corrected post-hoc test.

### Cell Lysis and Digestion

To detach adherent cells, media was removed, cells were briefly washed with sterile PBS and scraped off the plates utilising 1.5 ml ice cold PBS supplemented with cOmplete, EDTA-free Protease Inhibitor Cocktail Tablets (Roche, Mannheim, Germany). The cell suspension was transferred to a reaction tube and centrifuged at 4 ℃ and 16000 rpm for 5 min. The resulting pellet was washed three times in sterile PBS and stored at − 80 ℃ until further analysis. Cells were subsequently lysed in DIGE buffer (7 M Urea, 2 M Thiourea, 30 mM Tris Base, pH 8) homogenised for 3 min in an ultrasonic cycle of 30 s sonication and 30 s resting time on ice in a sonication bath. The lysate was centrifuged at 13000 g and 4 ℃ for 5 min and the supernatant was transferred in a novel reaction tube. The lysis step was repeated and the supernatant combined. Protein concentration was determined via Bradford assay and 20 µg of proteins were tryptically digested. For that, proteins were reduced and alkylated by subsequently adding 5 mM dithiothreitol for 30 min at 60 ℃ followed by 15 mM iodoacetamide for 30 min at RT. Digestion was carried out over night at 37 ℃ with a trypsin:protein ration of 1:40. Digestion was stopped by acidification. The peptide concentration was determined via amino acid analysis (May et al. 2021).

### Mass Spectrometric Measurements

The mass spectrometric measurements were performed on an Orbitrap Exploris™ 480 mass spectrometer (ThermoFisher Scientific, Bremen, Germany), online- coupled to a Vanquish Neo chromatography system (ThermoFisher Scientific, Bremen, Germany). 200 ng of peptides were injected and concentrated on a precolumn (PepMap Neo C18 Trap Cartridge 300 μm × 0.5 cm, particle size 5 μm, ThermoFisher Scientific, Bremen, Germany) and subsequently separated on an analytical column (DNV PepMapTM Neo, 75 μm × 150 mm, C18, particle size 2 μm, pore size 100 Å, ThermoFisher Scientific, Bremen, Germany). Peptides were separated at a flow rate of 400 nl/min and a solvent gradient of 1% B to 40% B (B: 84% acetonitrile, 0.1% formic acid) for 49 min. The separated peptides were ionised by ESI and injected into the mass spectrometer. The capillary temperature was set to 275 ℃ and the spray voltage to 1800 V. MS1 spectra were recorded in the range from 350 to 1400 m/z with a resolution of 120,000 at 200 m/z (normalised AGC-target 300%, maximum injection time 54 ms). For DIA analysis at MS2 level, 45 variable m/z windows were used and scanned at a resolution of 15000. Fragment ions were generated by HCD at a normalised collision energy of 30%. The mass spectrometry proteomics data have been deposited to the ProteomeXchange Consortium via the PRIDE (Perez-Riverol et al. 2025) partner repository with the dataset identifier PXD064335.

### Parallel Reaction Monitoring (PRM)

PRM measurements were conducted to confirm differential abundance of DBH, DDC and TH from DIA analysis. Comparable to DIA analysis, 200 ng of peptides were utilized for mass spectrometric measurements. Liquid chromatography (LC) was performed using the Vanquish Neo UHPLC system (ThermoFisher Scientific, Bremen, Germany). Samples were loaded on a precolumn (PepMap Neo C18 Trap Cartridge 300 μm × 0.5 cm, particle size 5 μm) and then separated on an analytical column (DNV PepMapTM Neo, 75 μm × 150 mm, C18, particle size 2 μm, pore size 100 Å). The peptides were separated at a flow rate of 400 nl/min and a solvent gradient of 1% B to 23% B (B: 84% acetonitrile, 0.1% formic acid) for 20 min and a subsequent increase to 42% for 10 min. The column was rinsed with 95% B for 5 min. The chromatography was online coupled to an Exploris480 mass spectrometer (ThermoFisher Scientific, Bremen Germany). Prior entering the mass spectrometer peptides were ionised by ESI. The capillary temperature was set to 275 ℃ and the spray voltage to 1800 V. MS1 spectra were recorded in the range from 375 to 1500 m/z with a resolution of 120000 at 200 m/z (normalised AGC-target 250%, maximum injection time 150 ms. An inclusion list of 30 precursors was included for PRM analysis see Table 1. MS2 scans were acquired at a resolution of 60,000, an isolation window of 0.8, an AGC-target of 1000% and a maximum injection time set to “Auto”. MS2 fragments were generated with a normalized collision energy of 30%. Data analysis was carried out in Skyline as described in the main manuscript. Results of PRM analysis can be found in supplementary figure S6-S10, followed by the corresponding inclusion list.

The data analysis was carried out as described in (Wulf et al. 2022). In brief, Tyrosine 3-monooxygenase (TH), Dopamine beta-hydroxylase (DBH) and Aromatic-L-amino-acid decarboxylase (DDC) were selected based on their results in our global proteomics study (Supplementary methods table X). The resulting peptide output was processed in an identical manner as our protein group output and peptides assigned to proteins of interest were examined for fold change, retention time and charge state. Evaluation of PRM data were performed using Skyline software (Pino et al. 2020) (version 24.1.0.199).

### Data Analysis

The generated DIA data (raw format) were analysed using the SpectronautPulsar software (version 16, Biognosys, Schlieren, Switzerland) and the directDIA option was selected for the analysis. The analysis settings were selected according to the manufacturer’s instructions with slight adjustments: Trypsin was added as digestive enzyme, carbamidomethylation on cysteine was chosen as fixed modification and oxidation on methionine as variable modification. The human reference proteome (accessed Uniprot 08.2022, 79,740 entries) (UniProt: the Universal Protein Knowledgebase in 2023 2023) was utilised to assign proteins. The non-normalized intensity values from the protein report were log2-transformed and normalized using an in house quality control script with a locally weighted scatter plot smoothing (LOESS) method (version 1.3). Subsequently, the normalised data were statistically analysed in Perseus (Tyanova et al. 2016). Significantly regulated proteins were determined by Student’s t-test and Benjamini Hochberg correction (q-value), whereby proteins with a q-value < 0.05 were considered statistically significant. Additionally, Gene Ontology enrichment analysis of significantly regulated proteins were performed on the basis of cellular components using Database for Annotation, Visualization and Integrated Discovery (DAVID).

Lastly, time-dependent protein abundance profiles of specific marker proteins were analysed. For that, the mean values of the label-free quantified (LFQ) intensities of the three biological replicates of daily harvested cells (day 1–day 6) were used and subtracted from the mean LFQ values of undifferentiated cells to determine their abundances in respect to the undifferentiated condition. Here line plots were created using R (R Core Team 2025) and ggplot2 (Wickham 2016).

## Results

Three cell subtypes can arise spontaneously in the SH-SY5Y neuroblastoma cell line, which are described as N- (neuroblastic), S- (Schwann-like) and I- (intermediate) subtypes, comprising different morphological characteristics. Differentiated (diff) N-type cells typically display elongated neurites, while S-type cells exhibit an epithelial-like trapezoidal structure (Kovalevich and Langford 2013; Acosta et al. 2009). However, these morphological differences are not present in undifferentiated (undiff) cells and thus molecular marker proteins are frequently utilised to decipher the true composition of a cell population and to determine successful differentiation. As a prerequisite, we aimed to verify whether all differentiation methods applied are capable of changing the composition of cell subtypes as well as to induce neurite outgrowth and extension.

### Differentiation of SH-SY5Y Cells does not Increase the Number of Neuroblastic Cells

The neuronal differentiation marker β-tubulin (TUBB) is a well-known marker for N-type cells (Dráberová et al. 1998; Lopez-Suarez et al. 2022) and vimentin (VIM), an intermediate filament involved in epithelial-mesenchymal transition (Gilles et al. 1999; Campos Cogo et al. 2020), tends to be increasingly expressed in S-type cells (Campos Cogo et al. 2020; Bell et al. 2013).

Thus, in a first step we aimed to investigate the abundance of all three cell types within undiff SH-SY5Y cells and the four differentiation strategies (Table 1), to identify the most suitable protocol for producing mature neurons via immunocytochemistry.

Diff (low RA, low RA/TPA, high RA/TPA, high RA) as well as the undiff SH-SY5Y cells showed positive protein expression for TUBB and VIM, whereby the N- and S-subtypes could be phenotypically classified on the basis of their immunoreactivity (see supplementary Fig. 1 A–E). Regarding the different differentiation methods, co-reactivity for both TUBB and VIM was frequently observed, potentially indicating a high abundance of the I-subtype, since they are thought to display properties of both N- and S-type cells.

**Fig. 1:**
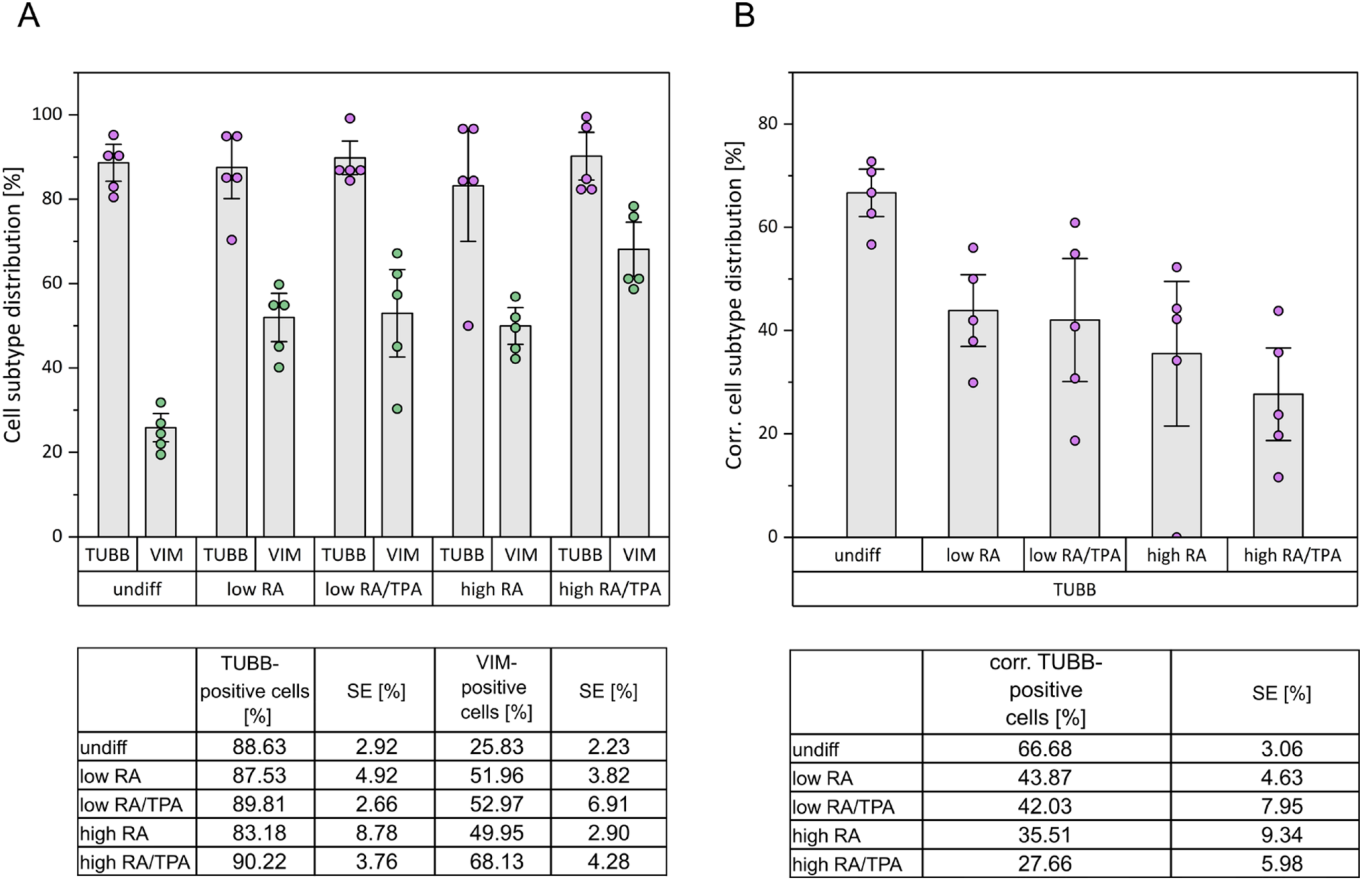
Distribution of neuroblastic (N)- and Schwann-like (S)-subtypes within the SH-SY5Y cell populations. The occurrence of the N- and S- subtypes should provide an overview of the distribution of neuron-like and non-neuronal cells within the different conditions. For this purpose, both the relative frequencies of βIII-Tubulin (TUBB)- and Vimentin (VIM)—positive or N- and S-type cells (**A**) and the proportion of TUBB- positive N-type cells corrected for I-type cells were considered (**B**). Relevant data are listed in tables below the respective diagrams. Undifferentiated cells (undiff) showed both the lowest incidence of S-type cells and the highest corrected proportion of N-type cells compared to the distributions resulting from the six-day differentiations (low (3% FBS) retinoic acid (RA), low RA/12-O-tetradecanoylphorbol-13-acetate (TPA), high(10% FBS) RA, high RA/TPA). When comparing the N- and S-type distributions within the four differentiations, it becomes clear that the cell population treated with a high FBS concentration and the combined use of RA and TPA (high RA/TPA) has the highest proportion of S-type cells and also the lowest proportion of N-type cells. The data represent mean values ± SEM (error bars) of approx. 430 to 460 analysed cells per differentiation method. Each data point corresponds to a 500 × 500 µm image section in which the labeled subtypes were counted

To obtain an overview of the distribution of cell subtypes within the undiff and diff SH-SY5Y cultures, the proportion of TUBB- and VIM-positive cells was determined (see supplementary table S1 for absolute and relative values and the statistical evaluation or Fig. 1A). Surprisingly, the proportion of N-type cells was highly consistent in all conditions including the undiff cells ranging from 83 to 90%. The highest percentage of N-type cells was observed in the high RA/TPA condition (90.22%) and the lowest in the high RA condition (83.18%). However, for S-type cells, greater variations were observed, with undiff cells exhibiting the lowest proportion of VIM-positive S-type cells (25.38%), while the highest proportion being observed in the high RA/TPA condition (68.13%). In a final step, intermediate (I)-type cells exhibiting characteristics of both subtypes were determined.

In order to ensure a stringent assessment of the distribution of N-type cells, the proportion of I-type cells was subtracted from the proportion of N-type cells resulting in a corrected frequency of N-type cells (see Fig. 1B). Counterintuitively, undiff cells showed the highest abundance of N-type cells. Utilising the corrected frequency of N-type cells, cells differentiated with a high FBS concentration (high RA, high RA/TPA) displayed a lower number of N-type cells compared to protocols using a low FBS concentration (low RA, low RA/TPA). Consequently, by examining the distribution of N- and S-type cells within the different conditions, it could be shown that undiff cells had both the highest percentage of N-type cells and the lowest incidence of S-type cells. This observation does not correspond to expectations, as literature states, that the use of differentiation agents such as RA and TPA generates homogeneous populations of N-type cells (Magalingam et al. 2020).

### A Low FBS Concentration Leads to an Increase in Neurite Length and Number of Neurite Projections

**Fig. 2:**
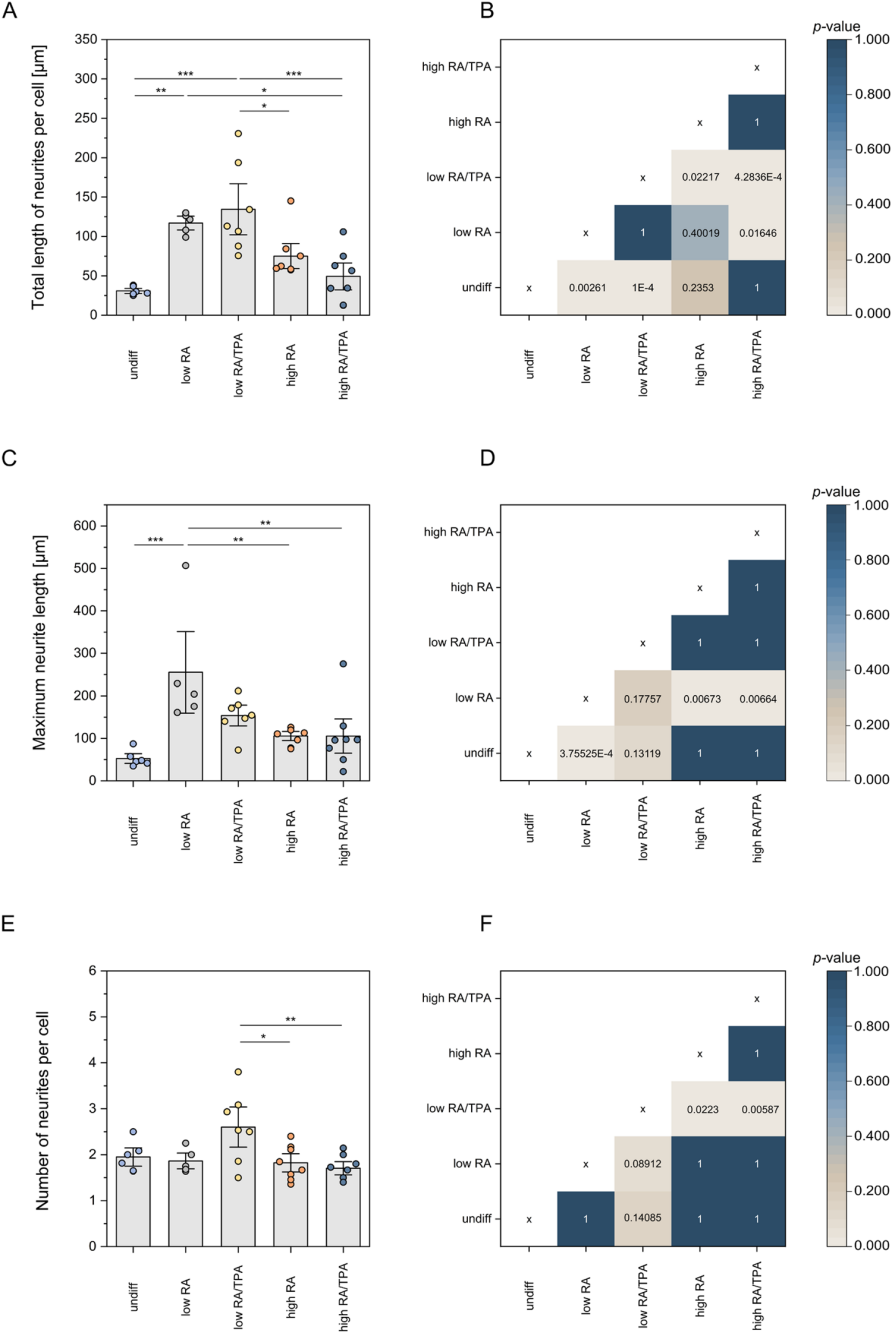
Neurite outgrowth parameters in undifferentiated (undiff) and differently differentiated (diff) SH-SY5Y cells. Panel A shows the total length of neurites per cell (in μm), panel C the maximum neurite length (in μm) and panel E the number of neurites per cell, which serves as an indicator of the degree of branching. The conditions low (3% FBS) retinoic acid (RA) and low RA/12-O-tetradecanoylphorbol-13-acetate (TPA) favoured a significant increase in neurite length, starting from the undiff state of the cells. A greater significant effect was achieved by the low RA/TPA-induced differentiation. Among all tested conditions, low RA treatment led to the strongest increase in maximum neurite length, showing significant differences compared to undiff cells, as well as to cells treated with high (10% FBS) RA and high RA/TPA. A high degree of branching could be stimulated by low RA/TPA. Each data point represents an image section in which fully traced neurites were quantified. Image sections were analysed until a total of 100 cells per condition had been evaluated. Data are shown as mean ± SEM (error bars). A one-way ANOVA was used to test the mean values for differences. The differences between the cells within the different populations were tested for significance using the Bonferroni-corrected post-hoc test (*p < 0.05; **p < 0.01; ***p < 0.001). For each of the three parameters assessed- the total length of neurites per cell (**B**), the maximum neurite length (**D**) and the number of neurites per cell (**F**)- the colour-coded matrices display pairwise comparisons between the different conditions. The colours of the individual cells represent the corresponding p-value, with lower p-values shown in light shades of beige. Overall, a low FBS concentration (low RA and low RA/TPA) resulted in improved neurite outgrowth

Mature neuronal cells are primarily defined by the expansion of their numerous, branched projections, which enable signal transmission and communication between neurons (Radio and Mundy 2008). Based on this, neurite length was determined for undiff cells and across all differentiation protocols. Tracking of neurite extensions was achieved by the enrichment of TUBB along neuritic projections and branches, which were visualised by immunofluorescence staining. With this, total length of neurites per cell, maximum neurite length and number of neurites per cell were assessed (see Fig. 2A, C, E and supplementary table S2).

Low FBS concentration (3%) led to a significant increase in the total length of neurites per cell after differentiation (*p* = 0.00261). A high FBS content (10%) in combination with RA/TPA displayed the lowest total neurite length (*p* = 0.000428). Regarding maximum length (see Fig. 2C), the differentiation agent RA combined with a low FBS concentration yielded the longest neurites. Significant differences were observed compared to high RA (*p* = 0.0067), high RA/TPA (*p* = 0.0066), and undiff cells (*p* = 0.000376). Additionally, a low FBS concentration combined with RA/TPA additives resulted in the highest number of neurites per cell (2.6 ± 0.29), although the difference was not statistically significant compared to undiff cells.

### A High FBS Concentration Favours the Abundance of S-type Marker Proteins

The results based on morphological analysis did not allow a clear recommendation as to which differentiation protocol promotes the presence of mature N-type cells and the loss of S-type cells.

Thus, in a next step, a mass spectrometry-based proteomic analysis was carried out (see supplementary table S3, for all quantified proteins, supplementary table S4 for significantly regulated proteins adjusted p-value < 0.05 and supplementary table S5 for significantly regulated marker proteins) to trace the expression levels of N- and S-type associated marker proteins for all differentiation approaches over the course of differentiation.

For S-type cells, the abundances of VIM, the S100 calcium binding protein A6 or calcyclin (CACYBP), beta-2-microglobulin (B2M) and the CD44 antigen (CD44) (La Quaglia and Manchester 1996; Acosta et al. 2009; Campos Cogo et al. 2020; Thirant et al. 2023) were considered (see supplementary figure S2, marked in green).

For B2M, VIM and CACYBP, a significant upregulation was observed in RA/TPA-diffcells compared to RA-diff cells (see supplementary figure S2A supplementary table S5). Furthermore, there appeared to be a correlation between a high FBS concentration and an increased abundance of VIM and CD44 (see supplementary figure S2B and supplementary table S5). A direct comparison between low RA and high RA/TPA-diff cells showed that all quantified S-type specific marker proteins were upregulated in the high RA/TPA condition (see supplementary figure S2C supplementary table S5). This data supports our morphological observation, that a high FBS concentration generally increases the abundance of S-type cells. Further, the classic differentiation approach utilising both RA and TPA also lead to increased abundance of said cell type (see supplementary table S5).

**Fig. 3:**
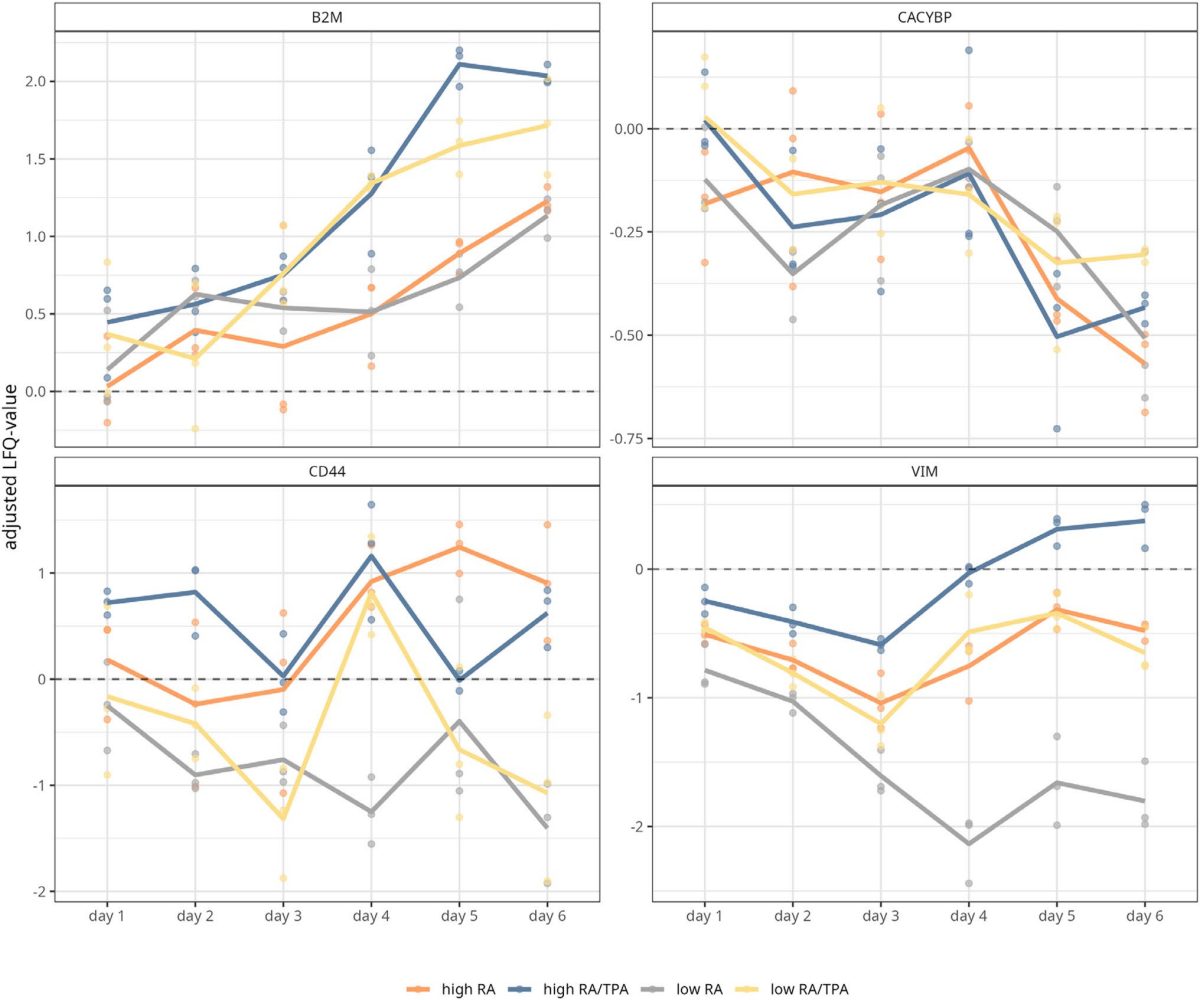
Time-dependent progression of Schwann-like (S)-type specific marker proteins in differentiated (diff) SH-SY5Y cells. The LFQ values of the S-type specific markers Vimentin (VIM), beta-2-microglobulin (B2M), S100 calcium binding protein A6 (CACYBP,C) and CD44 antigen (CD44) was tracked over the duration of differentiation (day 1 to day 6, (low (3%FBS) retinoic acid (RA) = grey line, low RA/12-O-tetradecanoylphorbol-13-acetate (TPA) = yellow line, high (10% FBS) RA = orange line, high RA/TPA = blue line)). For this purpose, the LFQ values of the markers in diff cells were normalised to those of undifferentiated (undiff) cells, resulting in corrected LFQ values. The dashed zero line represents the abundance of each respective marker in undiff cells. VIM shows a remarkable decrease in protein abundance in low RA-diff cells (grey line). The addition of RA (grey and orange lines) mediates a smaller increase in B2M abundance and a decrease in the abundance of CACYBP following the medium change on day 3

In order to gain a deeper insight into the dynamics of differentiation, the abundances of the above-discussed quantified marker proteins were determined in a time-dependent manner for the entire differentiation period (day 1—day 6). The abundance values of the undiff cells were utilised as a reference value and offset against the values of the diff conditions. In the following, S-type specific marker proteins with a linear progression were investigated (see Fig. 3). VIM initially showed a decrease in abundance in all conditions up to day 3 of differentiation (see Fig. 3A). However, after the addition of TPA on day 3, the abundance of VIM in high RA/TPA-diff cells increased, exceeding its abundance level in undiff cells on day 5 and day 6. The abundance of VIM also initially increased in low RA/TPA- and high RA-diff cells after medium exchange, but remained lower compared to undiff cells. Over the course of differentiation, a remarkable decrease in VIM abundance was only observed in the low RA-diff cells. In contrast, abundance of B2M generally increased with the duration of differentiation in all treatments (see Fig. 3).

Finally, the abundance of CACYBP was found to be lower compared to undiff cells for all differentiation approaches compared to undiff cells prior day 3 of differentiation (see Fig. 3). Nevertheless, after the addition of TPA on day 3, CACYBP abundance increased, exceeding the abundance level of undiff cells. In contrast, RA (low RA and high RA) mediated a decrease in the abundance of CACYBP.

In summary, our analyses confirmed that differentiation media containing a low FBS content led to a decreased abundance of S-type markers (except for B2M), and further that differentiation approaches utilising RA only led to lower abundances of S-type markers compared to approaches utilising a combined treatment.

For N-type cells, the abundances of TUBB and neurofilament light chain (NEFL) (Acosta et al. 2009) were evaluated (see supplementary figure S2, marked in purple). The N-type specific marker proteins however showed no consistency in their abundance in any of the comparisons.

### Confirmation of Successful Differentiation Based on General Neuronal Markers

In order to confirm successful neuronal differentiation and to assess the developmental stage of all conditions, the proteomic analysis was extended to a number of general neuronal markers. Further, the protein abundance of selected markers was compared between the differentiation methods (see data points shown in yellow in supplementary figure S3 and see supplementary table S5).

The neuronal markers included: enolase-2 (ENO2) (Hu et al. 2020), cytoskeletal components of mature neuronal cells such as microtubule associated protein 2 (MAP2), microtubule associated protein tau (MAPT), which plays an important role in axonal development (Maccioni and Cambiazo 1995), TIMP metallopeptidase inhibitor 2 (TIMP2), which acts as an anti-mitogenic signal, promoting neuronal differentiation and neurite outgrowth (Pérez-Martínez and Jaworski 2005), plasminogen activator, tissue type (PLAT), which serves as an indicator for axonal elongation during neuronal differentiation (Halakos et al. 2019) and neuronal pentraxin 2 (NPTX2), which plays a crucial role in the control of synaptic plasticity (Chapman et al. 2019). Additionally synaptic markers such as synaptic vesicle glycoprotein 2A (SV2A), synaptosome associated protein 25 (SNAP25) and syntaxin 1A (STX1A), which are involved in synaptic structure and neurotransmission (Agholme et al. 2010; Noor and Zahid 2017) were assessed. Further proteins that contribute to the stimulation of neurite outgrowth through interaction with the extracellular matrix, such as the neural cell adhesion molecule 1 (NCAM1), neural cell adhesion molecule 2 (NCAM2), integrin subunit alpha 1 (ITGA1)) (Bamdad et al. 2004; Murillo et al. 2017; Parcerisas et al. 2021), were investigated (see supplementary figure S1, A).

Lastly, we decided to include the extracellular glycoprotein elastin microfibril interfacer 1 (EMILIN1), a protein proposed to serve as a differentiation marker for SH-SY5Y cells (Murillo et al. 2017; Danussi et al. 2011), Nestin (NES), a marker of neuronal progenitor cells (Bott et al. 2019) and growth associated protein 43 (GAP43), a protein marker for undiff cells (Acosta et al. 2009).

Concerning the general neuronal markers investigated, none reached statistical significance (adjusted p-value < 0.05) between the different protocols, indicating that all differentiation methods are capable of producing mature neurons, except for NPTX2 which showed an increased abundance in low RA/TPA compared to high RA/TPA-diff cells. Similarly, NPTX2 and TIMP2 were found to be of higher abundance in low RA-diff cells compared to high RA-diff cells, although this difference was only found to be significant prior to p-value adjustment.

Both synaptic markers (SNAP25, STX1A) however, were found to be significantly increased by differentiation with RA compared to the high RA/TPA condition (see supplementary figure S3A). Confirmatively, NCAM1, NCAM2 and ITGA were also found to be significantly upregulated in RA differentiated cells (see supplementary figure S3A). In particular, NCAM2, which is essential in the cortex and hippocampus for proper neuronal differentiation, dendritic and axonal growth and synapse formation (Parcerisas et al. 2021) was found to be increased by sevenfold (see supplementary table S5). EMILIN1 was also highly abundant in RA-treated cells (approximately twofold, see supplementary figure S3A and see supplementary table S5). NES and GAP43, however, were both significantly upregulated by the sequential use of RA and TPA (see supplementary figure S3A and supplementary table S5).

Notably, NES was selectively upregulated at a high serum concentration, regardless of the selected additives (see supplementary figure S3B).

Overall, at the endpoint of differentiation, neuronal markers associated with mature neurons were increasingly upregulated in cells treated with RA only and a low FBS concentration. In particular, low RA-diffcells exhibited highly abundant neuronal markers associated with differentiation, neuritic growth and the presence of synapses.

The above-discussed neuronal markers were additionally examined in a time-dependent manner during differentiation in order to record dynamic changes in their abundance profiles. Markers displaying a clear expression pattern were selected for in depth examination (see Fig. 5). Again, the abundance values of the undiff cells were used as a reference value and offset against the values of the diff conditions (corrected LFQ value).

**Fig. 4:**
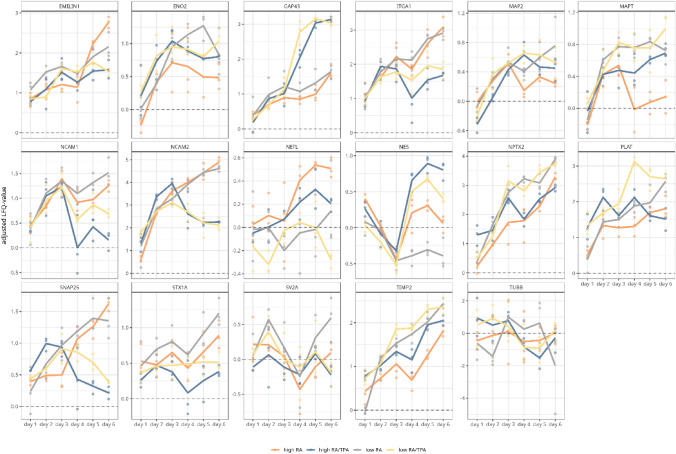
Time course dependent progression of protein abundance profiles of neuron-related markers. The LFQ values of the neuronal marker proteins are shown in relation to the individual days of differentiation (day 1 to day 6). For this purpose, the LFQ values of the markers in differentiated (diff) cells were normalised to those of undifferentiated (undiff) cells, resulting in corrected LFQ values. The dashed zero line represents the abundance of each respective marker in undiff cells. Line colors indicate the tested differentiation methods (grey = low (3% FBS) retinoic acid (RA), yellow = low RA/12-O-tetradecanoylphorbol-13-acetate (TPA), orange = high (10% FBS) RA and blue = high RA/TPA). The time-course of the protein abundance is shown for mature neuronal markers microtubule associated protein tau (MAP2), TIMP metallopeptidase inhibitor 2 (TIMP2), enolase-2 (ENO2), plasminogen activator, tissue type (PLAT) and neuronal pentraxin 2 (NPTX2). The synaptic markers synaptosome associated protein 25 (SNAP25) and neuronal pentraxin 2 NPTX2, neurite outgrowth-associated markers neural cell adhesion molecule 1 and 2 (NCAM1/2) and integrin subunit alpha 1 (ITGA1), the marker for neuronal precursor cells nestin (NES) elastin microfibril interfacer 1 (EMILIN) and Growth Associated Protein 43 (GAP43), which is associated with developing neurones. All tested differentiation methods generally induce an increase in the abundance of MAP2 and ENO2. Increased protein abundance levels of SNAP25, NCAM1/2, ITGA1 and EMILIN are observed under RA only conditions (grey and orange lines). Differentiation methods using low FBS concentrations (grey and yellow line) show a more pronounced increase in the abundance of NPTX2. The sequential use of RA and TPA (yellow and blue lines) mediates a greater increase in GAP43. Only low RA-diff cells (grey line) show a reduction in NES abundance level below the level of undiff cells after the medium change on day 3

On day 1 of differentiation both general neuronal markers (MAP2, ENO2) displayed a lower abundance in diff cells compared to the undiff counterpart. Until the media exchange on day 3 the abundance steadily increased reaching higher levels than in the undiff condition (see Fig. 4). The media exchange impacted the expression pattern in all differentiation protocols, with a particular decline observed in high RA and low RA conditions. However, by day 6, the final day of the differentiation period, low RA supplemented cells displayed the highest expression of MAP2 and low RA/TPA supplemented cells displayed the highest abundance of ENO2, again implying, that a low FBS concentration may be highly beneficial to promote neuronal differentiation. Confirmatively NPTX2 expression profiles displayed highest expression values for both low FBS-based differentiation protocols.

For the synaptic markers SNAP25, NCAM2 and ITGA1, a clear expression trend could be observed with all markers displaying the highest abundance in RA-treated cells, regardless of FBS content (see Fig. 4). A steady decline in expression was observed for both SNAP25 and NCAM2 after the addition of TPA on day 3, ITGA1 initially followed in a similar fashion, but expression increased again until the end of the differentiation period.

Regarding the expression of NES (see Fig. 4) all differentiation methods initially led to a decrease in protein abundance. Remarkably, an increase in abundance of NES was observed in high RA, low RA/TPA and high RA/TPA-diff cells after media exchange. Only low RA treatment ensured a level below the abundance of undiff cells GAP43 increased in abundance up to day 3 of the media exchange, regardless of the differentiation strategy (see Fig. 4). Interestingly, TPA supplemented media promoted a much higher increase in GAP43 abundance compared to RA only supplemented media.

In principle, the results obtained so far on the level of morphology, the presence of different cell types and the quantification of relative protein abundance of subtype-specific and neuronal markers confirm that all tested protocols are capable of mediating neuron-like properties. Nevertheless, the extent to which the treated cells acquired a mature neuronal phenotype varied across the different differentiation approaches.

### Differentiated SH-SY5Y Cells Display Unique Proteome Changes Depending on the Protocol

**Fig. 5:**
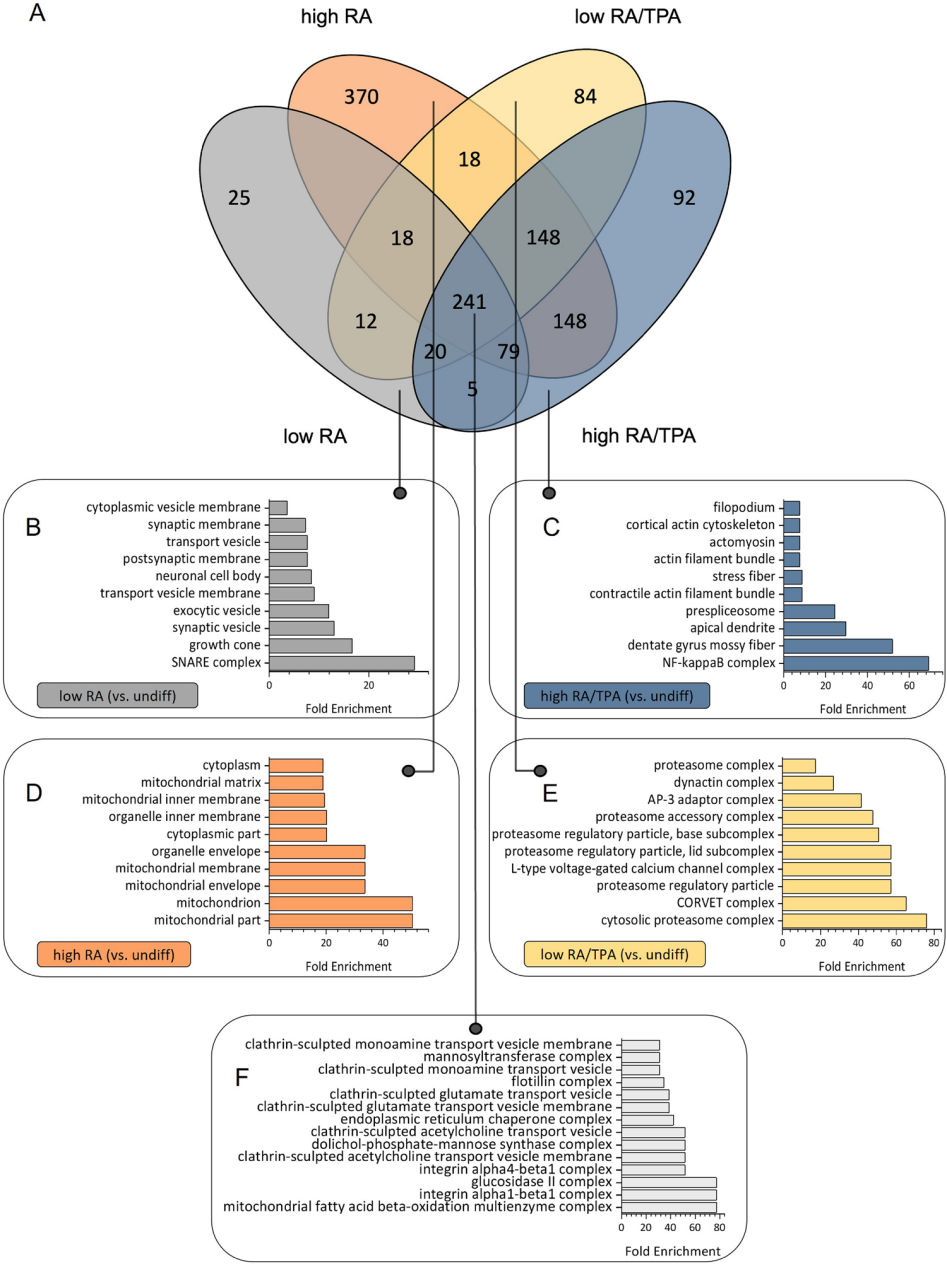
Gene ontology (GO) term analysis based on cellular components of differentially abundant protein groups in SH-SY5Y cells across various differentiation methods. Differentially upregulated proteins in differentiated cells compared to undifferentiated cells- either exclusively induced by low retinoic acid (RA), high RA/12-O-tetradecanoylphorbol-13-acetate (TPA), high RA, or low RA/TPA treatments, or jointly upregulated proteins, independent of the respective differentiation method- are graphically represented in the Venn diagram (A). Based on these proteins, or the genes coding for them, a GO analysis was performed to define enriched cellular components after differentiation (B-F). Here, the top 15 (F) or top 10 (B-E) GO terms with the highest fold enrichment values are listed. On the one hand, the GO analysis clarified the most important neuron-related components that were predominantly enriched by low RA. On the other hand, through the combined use of the differentiation agents RA and TPA, upregulated proteins were assigned to components indicative of tumour-specific, proliferative properties of the cell population

The assessment of general neuronal markers led to the impression, that a low FBS concentration may be favourable for the differentiation of SH-SY5Y cells to obtain mature neuronal cells with long neurite projections and an increased abundance of essential neuronal marker proteins. As showcased in recent studies, differentiation of SH-SY5Y cells leads to general changes in the proteome and is not only limited to neuronal specific proteins (Zhang et al. 2021; Barth et al. 2022; Eggers et al. 2025). Thus, an in-depth proteomic characterisation was carried out to gain insights into global- and protocol-specific molecular changes. For that:

1. proteins changing their abundance compared to undiff cells, regardless of the differentiation protocol (see Fig. 5A)
2. proteins changing their abundance with regards to the different differentiation strategies (see Fig. 5A)

were assessed. Initially, we identified 241 proteins that were commonly regulated in all differentiation strategies compared to undiff cells. We defined these proteins as the stable proteome of diff cells. GO Term analysis based on cellular components (CC) of identified proteins revealed a high abundance of cell adhesion receptors (see Fig. 5A, F), which generally play an important role in neurite development (Murillo et al. 2017). Furthermore, upregulated proteins were associated with vesicle formation and neurotransmitter release.

Next, we investigated proteins found to be specifically regulated in one condition compared to undiffcells. Here, 370 proteins were upregulated exclusively in high RA-, 84 in low RA/TPA-, 92 in high RA/TPA- and 25 in low RA-diff cells. These proteins were defined as differentiation strategy-specific proteins. Again, to obtain information about the functionality of these proteins, a GO analysis was performed (see Fig. 5B–F, and supplementary table S6).

Low RA-specific proteins were primarily associated with the SNARE protein complex, involved in membrane fusion of transport vesicles, as well as with the growth cone, controlling neurite extension (Squire 2009). Additional associations included the neuronal cell body and the postsynaptic membrane (see Fig. 5B). Notably, Dopa-decarboxylase (DDC), crucial for dopamine synthesis, was associated with multiple cellular components (e.g., synaptic vesicle, transport vesicle, see supplementary table S6), suggesting a potential differentiation into a dopaminergic phenotype induced by low RA.

Proteins that were identified as upregulated exclusively in the high RA condition were predominantly associated with mitochondrial proteins (mitochondrial part, mitochondrion, mitochondrial envelope, etc., see Fig. 5D). One interesting term that was exclusively associated with high RA/TPA supplementation is related to nuclear factor kappa B (NF-κB) activity (see Fig. 5C). NF-κB is a transcription factor whose activity promotes both tumour cell proliferation and epithelial-mesenchymal transition, which facilitates distant metastasis (Xia et al. 2014). In addition, upregulated proteins were assigned to splicing components whose activities are also associated with tumour cell proliferation (Ivanova et al. 2023), potentially indicating that high RA/TPA treatment may favour the cancer-like properties of SH-SY5Y cells. Nevertheless, several upregulated proteins could be associated with neuronal development as well (dentate gyrus mossy fiber, apical dendrite and components of the actin cytoskeleton).

A low FBS concentration and the combined use of the differentiation agents RA and TPA led to an increased abundance of proteasome-associated components (e.g., cytosolic proteasome complex, proteasome regulatory particle, proteasome regulatory particle, lid subcomplex, see Fig. 5E). Proteasomes play a crucial role in maintaining protein homeostasis, especially in rapidly proliferating cancer cells (Chen et al. 2021). However, cell components associated with neuronal cell excitability and neurotransmission (e.g., L-type voltage-gated calcium channel complex) were found to be enriched as well. Hence, in line with previous analyses, we concluded that the low RA/TPA treatment may induce a heterogeneous cell population with the characteristics of both proliferative and developing neuronal cells.

Overall, the GO analysis enabled us to propose several hypotheses regarding the effect to the different differentiation strategies:

- The combination of the differentiation agents RA and TPA induces a phenotype characterised by proliferative, tumour-specific properties, regardless of the FBS concentration.
- The differentiation agent RA primarily induces molecular responses associated with the development of a specific neuronal phenotype.
- In particular, low RA appeared to mediate a mature neuronal phenotype, as the main enriched components were associated with synapse formation, neurite outgrowth and induction of a dopaminergic neurotransmitter system.

### No Applied Differentiation Method Leads to a Defined Neuronal Subtype

**Fig. 6:**
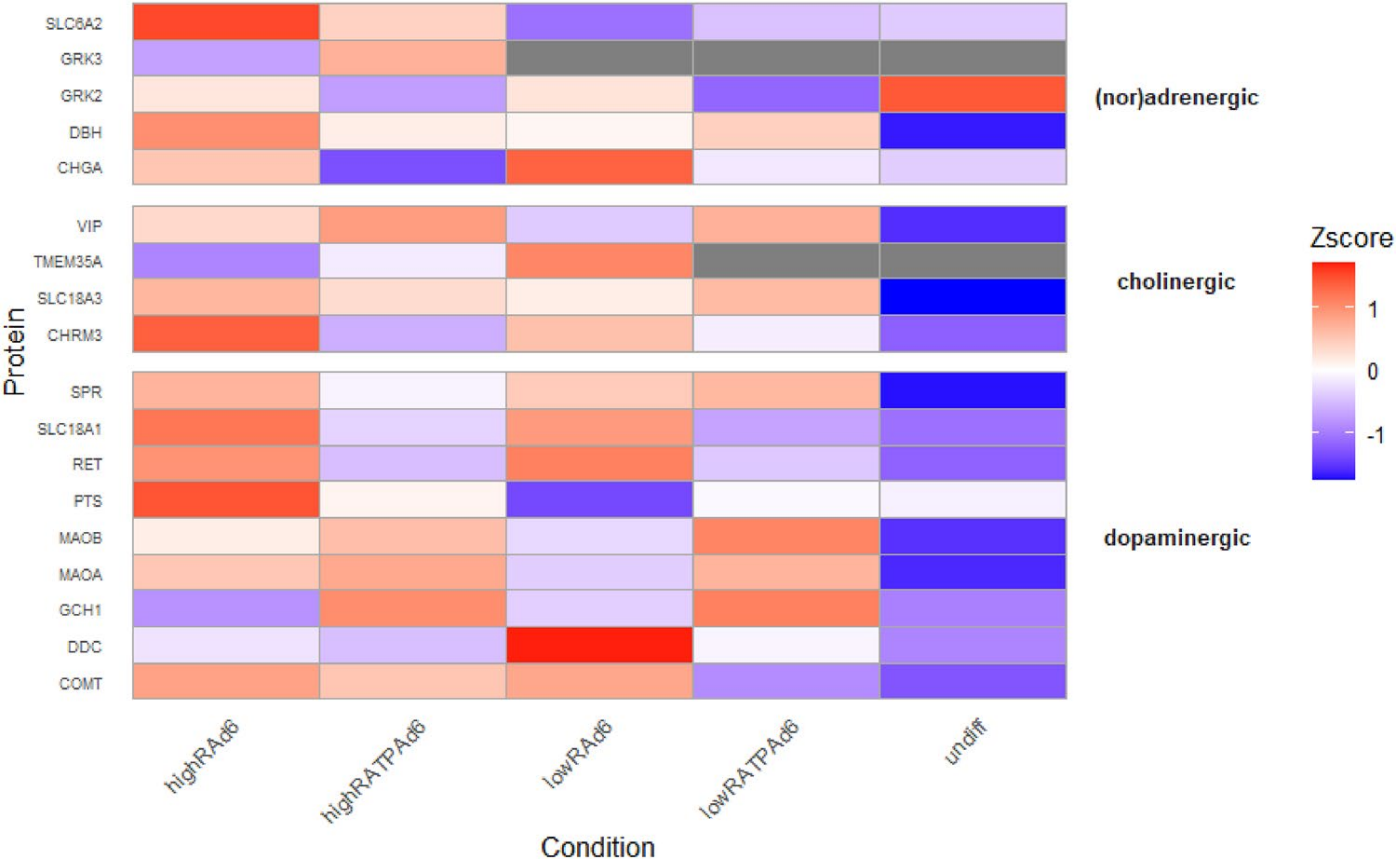
Heat map displaying the intensity of marker proteins for (nor) adrenergic, cholinergic and dopaminergic neuronal cells in undifferentiated (undiff) and fully differentiated (diff) (d6) SH-SY5Y cells diff either with high or low FBS (10% or 3%) and trans-retionoic acid (RA) or RA in combination with 12-O-tetradecanoylphorbol-13-acetate (TPA). High protein intensities are marked in red and low intensities in blue, grey indicates that the protein could not be identified in the respective condition. It is evident that low RA differentiation results in the highest abundance of DOPA-Decarboxylase (DDC), an essential protein in dopamine synthesis

Since the global comparison of all differentiation methods already indicated that a low FBS content and differentiation with RA only may favour a dopaminergic subtype, we continued to analyse the effect of the four differentiation approaches in relation to specific neuronal subtypes. For that, the abundances of identified protein markers specific for (nor) adrenergic, cholinergic or dopaminergic neurons were assessed in fully diff, as well as in undiff cells (see Fig. 6). It was evident that LFQ values of all subtype markers were found to be lowest in undiff SH-SY5Y cells, except for the two (nor) adrenergic markers beta-adrenergic receptor kinase 1 and 3 (GRK2, GRK3). When comparing the differentiation methods, the four (nor) adrenergic markers GRK2, sodium-dependent noradrenaline transporter (SLC6A2), dopamine beta-monooxygenase (DBH) and chromogranin-A (CHGA) (Swanson and Hartman 1975) were most abundant in SH-SY5Y cells diff under high RA conditions for 6 days. However, despite high overall abundance in this condition, the highest LFQ values for CHGA could be detected in low RA-diff cells. Confirmatively, time-course analyses revealed that CHGA levels continuously increased in low RA- diff cells, whereas CHGA levels steadily declined in cells diff with high RA/TPA over the course of the differentiation period (see supplementary figure S4).

The expression pattern of the four cholinergic markers (Ahmed et al. 2019), vasoactive intestinal peptide (VIP), novel acetylcholine receptor chaperone (TMEM35A), vesicular acetylcholine transporter (SLC18A3) and muscarinic acetylcholine receptor M3 (CHRM3) were not exclusively found to be highest in one differentiation condition. While the differentiation in high RA medium for 6 days resulted in a comparable expression pattern for all assessed markers, TMEM35A showed the highest abundance after 3 days of high RA differentiation (see supplementary figure S5), whereas the expression of VIP was found to be highest after RA/TPA differentiation for 6 days, regardless of the FBS concentration. Concerning dopaminergic markers, again no conclusive pattern could be observed. The main markers for dopaminergic neurons tyrosinse-3-monooxygenase (TH), D(1A) dopamine receptor (DRD1), D(2) dopamine receptor (DRD2) and sodium-dependent dopamine transporter (DAT) could not be identified in any of the samples. To elucidate whether the expression levels of TH may be under the limit of detection for global proteomics approaches, we additionally conducted parallel reaction monitoring of selected TH peptides (see supplementary figure S6-S8, as well as selected DDC and DBH peptides (see supplementary figures S9–S10). As positive control, we utilized a SH-SY5Y cell line overexpressing TH, kindly provided by Christine Klein (Prasuhn et al. 2017). Indeed positive signals for TH peptides could solely validated in the overexpressing cell linewhereby peptide signals for DDC and DBH were found to be present in all sample types, however with varying intensities.

Thus, relied on markers indicative for early events in DA metabolism such as GTP cyclohydrolase 1 (GCH1), 6-pyruvoyl tetrahydrobiopterin synthase (PTS) and Sepiapterin reductase (SPR) (Nishioka et al. 2022). In addition, proteins involved in the direct synthesis of DA (DDC), in the degradation (amine oxidase [flavin-containing] A/B (MAOA, MAOB), catechol O-methyltransferase (COMT)) and also in the vesicular transport of DA (solute carrier family 10 member 4 (SLC10A4) and chromaffin granule amine transporter (SLC18A1) were analysed in detail (Korecka et al. 2013; Meiser et al. 2013; Lohoff et al. 2019; Hobson et al. 2022). Here, a combined treatment of cells with RA followed by TPA seemed to favour the expression of the markers MAOA and MAOB, regardless of serum concentration. In contrast, DDC was found to be of highest abundance in cells diff under low serum condition and especially in cells diff with RA only.

### Differentiation with RA Only and a Low FBS Concentration Favours a Dopaminergic Entity

Since SH-SY5Y cells are commonly utilised as a model for Parkinson’s disease, we aimed to gain a deeper insight into the distribution of dopaminergic markers. For that, we directly compared the different differentiation strategies via a relative quantification approach to determine if any dopaminergic markers were found to be significantly enriched across the different conditions. We further validated the presence of dopamine in all differentiation approaches (see supplementary figure S11).

Our analysis revealed that the ret proto-oncogene (RET) (Airaksinen and Saarma 2002; Barth et al. 2022) and SLC18A1 (Lohoff et al. 2019) were found to be of higher abundance in low and high RA-treated cells compared to cells treated with RA/TPA (see supplementary figure S3, A). In contrast, GCH1 was found to be of higher abundance in high RA/TPA-diffcells compared to cells diff with high RA only (see supplementary figure S12A and supplementary table S5). Further, none of the markers were found to be differential between low RA/TPA and high RA/TPA treatment (see supplementary figure S12B and supplementary table S5).

When comparing the most widely used differentiation method in the literature (high RA/TPA) with the low RA condition, it was evident that DDC, RET and SLC18A1 were significantly enriched in low RA-diff cells, while GCH1 and MAOB were upregulated in high RA/TPA-conditions (see supplementary Fig. 12C and supplementary table S5).

**Fig. 7:**
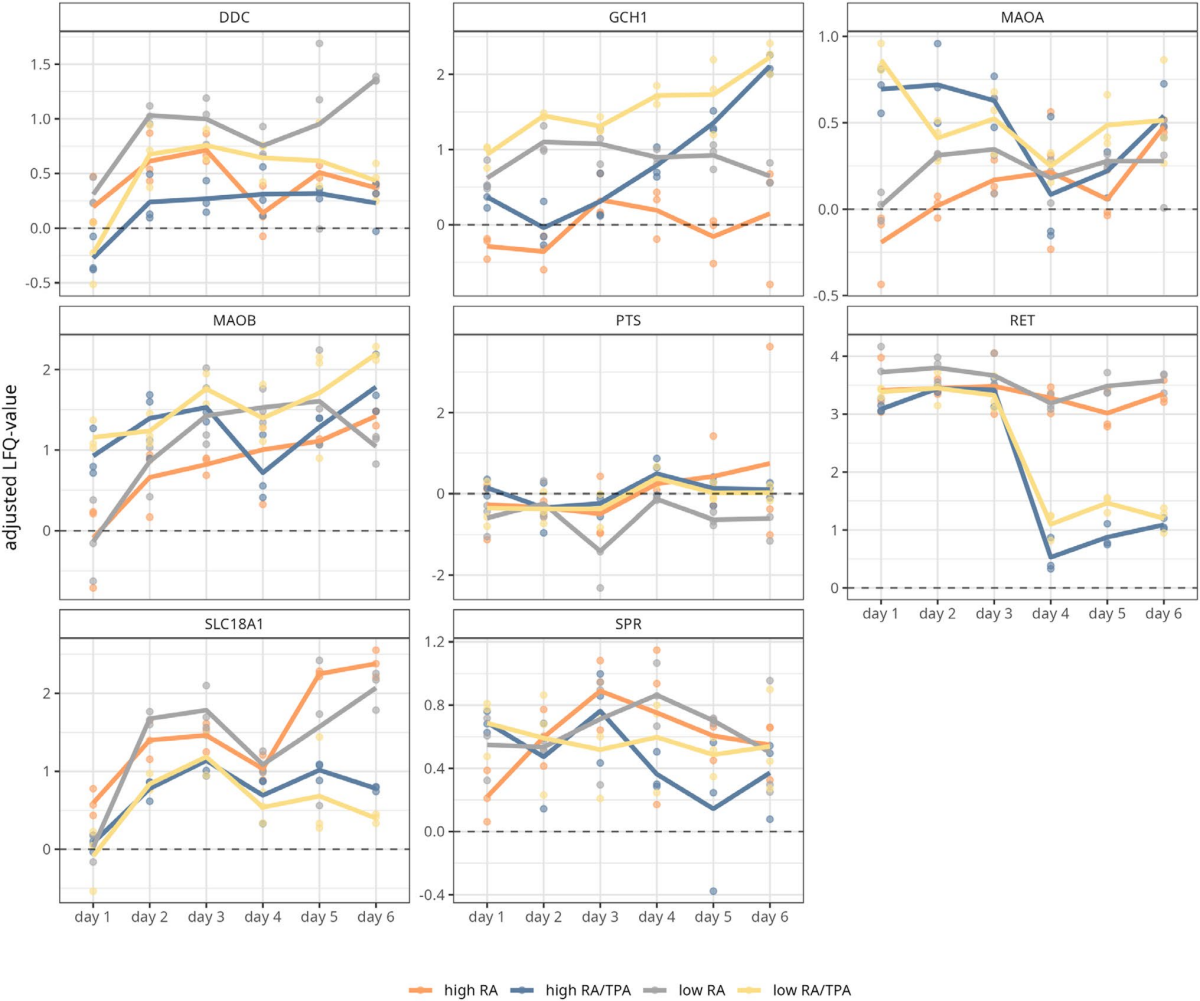
Time-dependent progression of dopaminergic marker proteins in differently differentiated SH-SY5Y cells. The protein abundance of the dopaminergic markers (DOPA-Decarboxylase (DDC), GTP cyclohydrolase 1 (GCH1) amine oxidase [flavin-containing] A (MAOA), amine oxidase [flavin-containing] B (MAOB), 6-pyruvoyl tetrahydrobiopterin synthase (PTS), ret proto-oncogene (RET), chromaffin granule amine transporter (SLC18A1), and Sepiapterin reductase (SPR*)*) was tracked across the differentiation period (day 1 to day 6). For this, mean LFQ values of three biological replicates of daily harvested cells were used. LFQ values of the marker proteins of the differently differentiated cells (low trans-retinoic acid *RA* grey line, low RA/12-O-tetradecanoylphorbol-13-acetate *TPA* yellow line, high *RA* orange line, high RA/TPA = blue line) were compared to those of undifferentiated cells. The dashed zero line represents the protein abundance of the respective marker in undifferentiated cells

To determine abundance changes of dopaminergic markers over the entire differentiation period, and thus to elaborate the time point from which a dopaminergic phenotype can be assumed, protein abundance profiles were monitored in a time-dependent manner and set in relation to those of undiff cells (see Fig. 7 and supplementary table S5).

Confirmatively to our prior observations, a low FBS concentration favoured the expression of DDC since its levels were found to be consistently higher over the complete course of differentiation, compared to undiff cells (see Fig. 7 supplementary table S5). In particular, low RA treatment resulted in the highest expression of DDC at the end point of differentiation. In contrast, cells diff with a high FBS concentration (high RA and high RA/TPA) displayed lower DDC expression levels than undiff cells on day 1, with only a moderate increase over the course of differentiation. The abundance level of RET and SLC18A1 were both closely linked to RA supplementation, regardless of FBS concentration. However, after TPA addition on day 3 of differentiation, the abundance of RET and SLC18A1 rapidly declined.

The expression of GCH1 was mainly favoured by the combined treatment of RA/TPA regardless of FBS concentration. Nonetheless, utilising a low FBS concentration led to a higher GCH1 abundance from day 1 of the differentiation period. Thus, it can be summarized that, among all differentiation protocols assessed, a low FBS concentration and supplementation with RA only favours a dopaminergic subtype.

## Discussion

The SH-SY5Y cell line is one of the most widely utilised cell lines in neuroscience, spanning studies on neurotoxicity, neuronal development and neurodegeneration. For the majority of research questions, inducing a mature neuronal phenotype is of highest interest.

However, so far, no systematic comparison of the most utilised differentiation approaches on the level of proteins has been performed. Successful differentiation into one neuronal subtype was instead preferentially confirmed via immunocytochemistry, western blot or on the level of RNA. First studies analysing the proteome of undiff and diff SH-SY5Y cells reported a diverse abundance of several neuronal marker proteins and most often the lack of defined dopaminergic markers, such as tyrosine hydroxylase or dopamine transporters.

With our mass spectrometric approach, we were able to formulate general statements regarding the most widely applied differentiation approaches:

1. Differentiation with RA induces a more mature neuronal phenotype in SH-SY5Y cells
2. Differentiation with RA and TPA favours the procurement of a heterogeneous cell population
3. Reduced FBS concentration contributes to the promotion of neuronal differentiation

### Differentiation with RA Induces a More Mature Neuronal Phenotype in SH-SY5Y Cells

Although the differentiation strategies examined here did not produce homogeneous cell populations, RA-treated cells showed a significantly reduced abundance of S-type specific marker proteins (see supplementary figure S2, A–C and Fig. 3B–c), potentially indicating a gradual decrease of non-neuronal cells. Confirmatively proteins uniquely upregulated in response to RA treatment only were found to be associated with the formation of neuritic projections and synaptic transmission. This assumption is consistent with the increased expression of neuronal marker proteins such as NCAM1, NCAM2, ITGA1, EMILIN1, SNAP25 and STX1A in RA-treated cells (see supplementary figure S3A and supplementary table S5), all of which showed progressive upregulation during the differentiation period. The upregulation of ITGA1, NCAM1 and NCAM2 correlates with synaptogenesis, which plays an essential role in the organisation of the cytoskeleton and the morphological development of neurons (Murillo et al. 2017; Parcerisas et al. 2021). Similar results were obtained by Zhang et al. in a mass spectrometry-based quantitative analysis using tandem atomic mass tags (Zhang et al. 2021). In that study, the proteome profile of SH-SY5Y cells was investigated during a seven-day RA treatment and an upregulation of proteins associated with the assembly of neuronal projections was detected.

### Differentiation with RA and TPA Favours the Procurement of a Heterogeneous Cell Population

Previous studies have shown that TPA belongs to the class of potent tumour-promoting agents and exerts mitogenic effects in some in vitro models (Blumberg 1980). The effects of TPA and related phorbol esters are complex and the morphological and biochemical changes associated with TPA treatment vary depending on the cell system under investigation (Påhlman et al. 1981). On the one hand, an inhibitory effect of TPA on induced differentiation was found in cultured cells (Rovera et al. 1977; Fibach et al. 1979). A reversible inhibition of neurite formation was also induced by TPA in mouse neuroblastoma cells (Ishii et al. 1978). On the other hand, induced differentiation of SH-SY5Y cells has also been observed by the development of neuron-like projections, increased expression of neuronal markers and a decrease in the proliferation rate (Påhlman et al. 1981).

Furthermore, the results regarding an induced dopaminergic phenotype are also controversial.

While the sequential use of RA and TPA is thought to efficiently mediate mature neuronal cells with dopaminergic properties (Xie et al. 2010; Xicoy et al. 2017; Kovalevich and Langford 2013; Presgraves et al. 2004) and supported by positive immunoreactivity for TH, DRD1 and DRD2 receptors, DAT and SLC18A1 (Presgraves et al. 2004), other studies observed a decrease in expression profiles of TH, DDC and SLC18A2 (VMAT2) compared to undiff controls (Filograna et al. 2015).

In this work, the characterisation of specific proteomic changes indicated that RA/TPA treatment induced a heterogeneous cell population containing both proliferative/tumour-specific as well as neuronal properties (see Fig. 5). High RA/TPA-specific proteins were primarily associated with NF-κB activity. NF-κB is a transcription factor whose activity plays a key role in the development and metastasis of cancer in humans. Its activity promotes both tumour cell proliferation and epithelial-mesenchymal transition, which facilitates distant metastasis (Xia et al. 2014).

In addition, upregulated proteins were assigned to splicing components, which are connected to an increased proliferation of tumour cells, invasion and metastasis and thus the promotion of tumour progression (Ivanova et al. 2023). To conclude, our study indicates that the sequential use of RA and TPA favours cancer-like properties of the SH-SY5Y cell line as a neuroblastoma derivative. In line with this, within the listed comparisons of the differentiation conditions, S-type specific markers were significantly more abundant when both additives RA and TPA were used (see supplementary figure S2). This could be further supported by evaluating the expression of S-type markers CACYBP and B2M over the entire course of differentiation, both of which showed an increased slope following the addition of TPA (see Fig. 3). Additionally, GAP43, a marker for undiff neural crest-derived cells (Acosta et al. 2009), was consistently highly abundant in RA/TPA-diff cells and its time-dependent expression showed a particular increase after TPA supplementation on day 3 (see Fig. 4).

Thus, it can be assumed that RA/TPA-induced differentiation predominantly favours the maturation of non-neuronal, epithelial cells, whereas neuronal cells present, may not fully develop into full mature neurons.

### Reduced FBS Concentration Contributes to the Promotion of Neuronal Differentiation

The morphological analyses of SH-SY5Y cells treated with a reduced FBS concentration initially revealed a slightly increased proportion of neuron-like N-type cells (see Fig. 1 and supplementary figure S1). Furthermore, it could be demonstrated that the cells diff with a low FBS concentration showed an overall improved neurite outgrowth (see Fig. 2). Neurite growth occurs as a result of differentiation of progenitor cells into a terminal neuronal phenotype and is a key determinant of neuronal connectivity (Radio and Mundy 2008). Neurite length directly correlates with the degree of differentiation (Halakos et al. 2019). Confirmatively, our results of the quantification of neurite outgrowth indicated that a reduced FBS concentration favours neuronal differentiation. In addition to these morphological changes, proteomic analyses demonstrated that a reduction in FBS concentration does affect the abundance profiles of markers associated with neurite outgrowth and synaptic plasticity. Notably, PLAT, which is secreted at the growth cone in extending neurites (Halakos et al. 2019), as well as NPTX2 and neurogranin (NRGN), which are involved in the modulation of synaptic plasticity (Chapman et al. 2019; Hwang et al. 2021), showed increased abundances under low FBS conditions. Similar conclusions were drawn in a study analysing gene expression changes following serum deprivation in diff SH-SY5Y cells (Thomson et al. 2022). Extracellular signals that function as chemotactic or directional cues, play an important role in initiating neurite formation and elongation (Clagett-Dame et al. 2006). The growth factors or neurotrophic factors contained in the FBS (Anderson et al. 2004) could act as such cues and initiate directional growth. If these types of signalling proteins are present in reduced concentrations in the differentiation medium, neuritic processes may need to expand further in order to reach the chemical stimuli. Thus, a reduced FBS concentration may contribute to the organisation of neuronal structures, thereby promoting synaptic networking and the maturation of a neuronal system. This assumption is consistent with the differentially abundant protein perilipin 2 (PLIN2), which was significantly upregulated under low FBS concentrations, regardless of the choice of additives (see supplementary table S4). PLIN2 belongs to the PLIN proteins that associate with intracellular lipid droplets and regulate lipolytic activity. In addition, PLIN2 is thought to promote Wnt/β-catenin signalling and the expression of Wnt target genes (Liu et al. 2016). In the central nervous system, the Wnt signalling pathway controls neurite development and growth, synaptic function and neuronal plasticity, thereby regulating the formation and function of neuronal circuits (Rosso and Inestrosa 2013).

Within the differentiation strategies investigated, a low FBS concentration therefore appears to promote the maturation of neuron-like cells. Nevertheless, when using FBS in-cell culture media, it should generally be taken into account that it contains numerous components that can vary depending on the batch number. This, in turn, can have disruptive effects on gene expression and lead to phenotypic variations in-cell cultures (Thomson et al. 2022). From both scientific and ethical perspectives, use of FBS is being increasingly debated and the implementation of a suitable alternative (e.g., human platelet-derived platelet lysate) could be considered (Subbiahanadar Chelladurai et al. 2021).

### The low RA Differentiation Method Mediates a Mature Neuronal Phenotype with a Dopaminergic Character

Differentiation is thought to achieve a mature neuronal phenotype. However, as outlined in this study, different differentiation strategies result in varying morphological and molecular responses of neuronal precursor cells. As discussed, differentiation using a low FBS concentration and RA only, resulted in an upregulation of proteins associated with synaptic transmission, growth of neurite projections and particularly a dopaminergic neurotransmitter system (see Figs. 1,2,5,6 and 7). In addition, the S-type marker VIM, which is also thought to have a prominent role in carcinogenesis, displayed a remarkable decrease during differentiation exclusively in low RA-treated cells (see Fig. 3A). Furthermore, compared to the differentiation strategies tested here, solely treatment with low RA maintained NES abundance below the level of undiff cells (see Fig. 4). Since NES promotes proliferation in neuronal progenitor cells (Magalingam et al. 2020), its downregulation suggests that early stages of neuronal maturation have been overcome and the low RA-treated cells may be terminally diff.

Thus, differentiation with low RA may facilitate the transition from the undiff to the neuronal state while repressing tumour-specific properties. When comparing the differentiation conditions low RA and high RA/TPA, the majority of quantified neuronal markers, especially those associated with neuritic growth, synaptogenesis and neurotransmission, were significantly upregulated in low RA-treated cells (see Fig. 5 and supplementary table S6). With regard to the induction of a mature neuronal phenotype, the low RA differentiation method should therefore be preferred. However, the functionality of diff neuronal cells in terms of synaptic transmission should be further validated, e.g., by the patch-clamp technique (Accardi et al. 2016) or by calcium flux assays triggered by electrical field stimulation (Virdee et al. 2017), to strengthen the above-discussed statements.

Since SH-SY5Y cells are most commonly used to investigate neurodegeneration and neurodegenerative diseases, in particular PD, we aimed to decipher its potential to mimic characteristics of dopaminergic neurons.

A systematic review of scientific publications on SH-SY5Y cells utilised in PD research showed that out of 962 included publications, approximately 82% did not use any differentiation protocol, approximately 2% did not report differentiation status and approximately 16% did differentiate utilizing numerous protocols with various differentiation agents. This lack of standardisation highlights a critical issue, particularly since the extent to which this cell line exhibits a dopaminergic phenotype directly affects the model’s validity (Xicoy et al. 2017).

Given that the majority of studies did not confirm the presence of dopaminergic markers and that no clear consensus of an optimal differentiation strategy exists to date, we aimed to investigate the most widely used differentiation protocols ((RA and RA/TPA, low (3%) and high (10%) FBS) regarding their potential of enforcing a dopaminergic phenotype on the morphological and molecular level. However, none of the classic markers for dopaminergic neurons, such as TH, D(1A) DRD1, DRD2 and DAT could be identified in any of the samples. Although a few publications demonstrated the presence of said proteins in SH-SY5Y cells via Western Blot or immunocytochemistry (Presgraves et al. 2004; Khwanraj et al. 2015; Alrashidi et al. 2021), mass spectrometry-based analyses were unable to confirm their presence in either undiff or diff cells. Similarly, other proteomic investigations utilising SH-SY5Y cells did not report the presence of any of those marker proteins (Zhang et al. 2021; Eggers et al. 2025; Barth et al. 2022), although all of the above mentioned proteins are easily identified by mass spectrometry in brain tissue (Wulf et al. 2022), in iPSCderived human dopaminergic neurons (Cavarischia-Rega et al. 2024) and in SH-SY5Y cells overexpressing TH as validated in our PRM approach (see supplementary figure S6-S8). The identification of dopaminergic markers, particularly TH, in SH-SY5Y cells remains controversial, as findings vary greatly between studies and applied methods, raising questions as to whether SH-SY5Y cells may be highly diverse in their composition, depending on their source, cultivation and propagation in culture. Since the presence of dopamine in differentiated SH-SY5Y could be verified in a prior study utilizing a HPLC-based approach (Eggers et al. 2025), as well as in the current study by ICC staining of dopamine, one may assume that L-DOPA may be present in the cell culture media in particular in FBS, which may in turn prevent the expression of TH on the protein level, since it is not needed to convert L-tyrosine to L-DOPA to enable dopamine production. Unfortunately, no information on L-DOPA concentrations in FBS could be retrieved from the vendor, thus this hypothesis has to be further validated by measuring L-DOPA as well as other metabolites in the dopamine synthesis pathway or by complete serum deprivation to potentially induce TH expression.

To formulate a hypothesis as to which protocol may produce a dopaminergic-like subtype, we thus focused on other proteins involved in the dopaminergic pathway. Here, the marker DDC was always found to be significantly upregulated in low RA-diff cells (see supplementary figure S12 and supplementary table S5). This is also consistent with the time-course of DDC abundance (see Fig. 7), which showed a clear upward trend in low RA-differ cells. The decarboxylation of L-DOPA by DDC leads directly to the synthesis of DA (Meiser et al. 2013), which could be validated to be present on the level of ICC for all four differentiation strategies (see supplementary figure S11). This provides promising evidence for the induction of the dopaminergic neurotransmitter system by low RA in particular. Furthermore, the courses of MAOA and MAOB in low RA-diff cells tended to show reduced abundances compared to the differentiation methods tested here. Although it remains controversial whether specifically MAOA or MAOB contributes to dopamine degradation (Schapira, Anthony H.V., Lang A.E.T., Fahn S. 2010; Cho et al. 2021), one study attributed the reduced MAOA levels in diff SH-SY5Y cells to the synthesis and storage of DA (Magalingam et al. 2020).

In summary, our findings suggest that the differentiation strategy with a reduced FBS concentration and the addition of RA (low RA) may be preferred to treatments with an increased FBS concentration or the sequential use of the additives RA and TPA (high RA/TPA, low RA/TPA and high RA) for the mediation of mature neuron-like cells with a dopaminergic character. Nevertheless, the SH-SY5Y cells could not be considered as authentic dopaminergic neurons (Pingale and Gupta 2020). Compared to iPSC-derived human dopaminergic neurons, whose proteome solely contain dopaminergic marker proteins (Cavarischia-Rega et al. 2024), SH-SY5Y cells always do contain a mixture of markers indicative of several neuronal subtypes.

Further investigation on the abundance of all neuronal subtypes via ICC staining of marker proteins may help to estimate the ratio of neuronal subtypes, which cannot be achieved on the level of masss spectrometry, if no single cell approaches are followed. Further prolonged differentiation periods, or the utilisation of alternative media compositions e.g., neurobasal-media may be necessary to achieve a clear induction of a dopaminergic neurotransmitter system as demonstrated in several studies (Lopes et al. 2017; Constantinescu et al. 2007; Shipley et al. 2016).

## Conclusion

With our in-depth characterization of the four most commonly applied differentiation methods of SH-SY5Y cells, we were able to formulate that differentiation with RA induces a more mature neuronal phenotype in SH-SY5Y cells, whereby the combined use of RA and TPA favours a heterogeneous cell population, consisting of N- and S-type cells. Further, we were able to conclude that a reduced FBS concentration favours neuronal differentiation. Lastly, we demonstrated that regardless of the applied differentiation approach, the resulting neuronal cells express markers indicative of several neuronal subtypes, although low serum condition and the differentiation reagent RA seem to favour a dopaminergic identity. With this we clearly demonstrate that a prior characterisation of SH-SY5Y cells is indispensable to ensure that the model’s validity is appropriately aligned with the specific research question.

## Supplementary Information

Below is the link to the electronic supplementary material.

Supplementary file1 (RAR 8509 KB)

## Data Availability

All data analysed during this study are included in this published article and its supplementary information files.The mass spectrometry proteomics raw data have been deposited to the ProteomeXchange Consortium (http://proteomecentral.proteomexchange.org) via the PRIDE partner repository (Perez-Riverol et al. 2025) with the dataset identifier PXD064335.

## Abbreviation

TPA: 12-O-tetradecanoylphorbol-13-acetate
MAOA: Amine oxidase [flavin-containing] A
MAOB: Amine oxidase [flavin-containing] B
B2M: Beta-2-microglobulin
GRK: Beta-adrenergic receptor kinase
CACYBP: Calcyclin
COMT: Catechol O-methyltransferase
CD4: 4CD44 antigen
SLC18A1: Chromaffin granule amine transporter
CHGA: Chromogranin-A
DRD1: D(1A) dopamine receptor
DRD2: D(2) dopamine receptor
Diff: Differentiated
*DDC*: Dopa-decarboxylase
DA: Dopamine
DBH: Dopamine beta-monooxygenase
EMILIN1: Elastin microfibril interfacer 1
ENO2: Enolase 2
FBS: Foetal calf serum
GAP43: Growth associated protein 43
GCH1: GTP cyclohydrolase 1
ITGA1: Integrin subunit alpha 1
I-type: Intermediate-type
LFQ: Label-free quantification
MAP2: Microtubule associated protein 2
CHRM3: Muscarinic acetylcholine receptor M3
NES: Nestin
NCAM1: Neural cell adhesion molecule 1
NCAM2: Neural cell adhesion molecule 2
N-type: Neuroblastic-type
NEFL: Neurofilament light chain
NPTX2: Neuronal pentraxin 2
TMEM35A: Novel acetylcholine receptor chaperone
PD: Parkinson’s disease
PLAT: Plasminogen activator, tissue type
RET: Ret proto-oncogene
S-type: Schwann-like-type
SLC6A2: Sodium-dependent noradrenaline transporter
SLC10A4: Solute carrier family 10 member 4
SV2A: Synaptic vesicle glycoprotein 2A
SNAP25: Synaptosome associated protein 25
STX1A: Syntaxin 1A
TIMP2: TIMP metallopeptidase inhibitor 2
RA: Trans-retinoic acid
TH: Tyrosinse-3-monooxygenase
Undiff: Undifferentiated
VIP: Vasoactive intestinal peptide
SLC18A3: Vesicular acetylcholine transporter
VIM: Vimentin
TUBB: βIII-Tubulin
